# Exposure to family planning messages and contraceptive use among women of reproductive age in sub-Saharan Africa: a cross-sectional program impact evaluation study

**DOI:** 10.1038/s41598-022-22525-1

**Published:** 2022-11-07

**Authors:** Duah Dwomoh, Susan Ama Amuasi, Emefa Modey Amoah, Winfred Gborgbortsi, John Tetteh

**Affiliations:** 1 Department of Biostatistics, School of Public Health, University of Ghana, Accra, Ghana; 2 Department of Physician Assistantship, School of Medicine and Health Sciences, Central University College, Accra, Greater Accra Ghana; 3 Department of Population, Family and Reproductive Health, School of Public Health, University of Ghana, Accra, Ghana; 4 School of Geography and Environmental Science, University of Southampton, Highfield, SO17BJ UK; 5 Department of Community Health, University of Ghana Medical School, University of Ghana, Accra, Ghana

**Keywords:** Health care, Medical research

## Abstract

Many women of reproductive age in sub Saharan Africa are not utilizing any contraceptive method which is contributing to the high burden of maternal mortality. This study determined the prevalence, trends, and the impact of exposure to family planning messages (FPM) on contraceptive use (CU) among women of reproductive age in sub-Saharan Africa (SSA). We utilized the most recent data from demographic and health surveys across 26 SSA countries between 2013 and 2019. We assessed the prevalence and trends and quantified the impact of exposure to FPM on contraceptive use using augmented inverse probability weighting with regression adjustment. Sensitivity analysis of the impact estimate was conducted using endogenous treatment effect models, inverse probability weighting, and propensity score with nearest-neighbor matching techniques. The study involved 328,386 women of reproductive age. The overall prevalence of CU and the percentage of women of reproductive age in SSA exposed to FPM were 31.1% (95% CI 30.6–31.5) and 38.9% (95% CI 38.8–39.4) respectively. Exposure to FPM increased CU by 7.1 percentage points (pp) (95% CI 6.7, 7.4; p < 0.001) among women of reproductive age in SSA. The impact of FPM on CU was highest in Central Africa (6.7 pp; 95% CI 5.7–7.7; p < 0.001) and lowest in Southern Africa (2.2 pp; 95% CI [1.3–3.0; p < 0.001). There was a marginal decline in the impact estimate among adolescents (estimate = 6.0 pp; 95% CI 5.0, 8.0; p < 0.001). Exposure to FPM has contributed to an increase in CU among women of reproductive age. Programs that are geared towards intensifying exposure to FPM through traditional media in addition to exploring avenues for promoting the appropriate use of family planning method using electronic media remain critical.

## Introduction

Currently, the global estimates of maternal mortality (MM), although indicative of improvements, remain unacceptably high^1,2^. In 1987, the Safe Motherhood Initiative (SMI) was launched as an initiative to enhance the quality of maternal health. The focus of the initiative was channeled to people living in low-and-middle-income countries (LMICs). The SMI envisages that in making an impact to minimize MM, all women must have access to essential health services including family planning^3^. However, many women of reproductive age are not utilizing any contraceptive method which is contributing to the high burden of MM. It has been established that a considerable number of MM would be avoided if the contraceptive prevalence rate (CPR) increased, and the unmet need for FP decreased^4^. Contraceptive use reduces the risk of unplanned pregnancy^5,6^, and provides substantial social and economic benefits including improved educational and employment opportunities^7^. Additionally, contraceptive use reduces unwanted fertility which is a major public health problem in developing countries^4^.

Globally, 172 million women are currently not using any method of contraception even though they desire to avoid pregnancy^8^. In 2013, Darroch and colleagues found that the unmet need for modern contraceptives in sub-Saharan Africa (SSA) was 60% of the 89 million population^9^. The non-use of contraceptives varies across the continent. However, unmet needs, health concerns, infrequent sex, opposition from others, lack of knowledge, and other less reported factors are the key barriers hindering contraceptive use (CU)^10,11^.

In low and middle-income countries, it is estimated that a quarter of women of reproductive age wish to avoid pregnancy but are not utilizing effective contraceptive methods^7^. Among women aged 15–49 years in 47 developing countries, this was observed to be higher at an average of 40.9% who needed contraception but are not using any technique^12^.

All sexually active women should be informed about their risk of becoming pregnant as well as the many techniques available to prevent unplanned or unintended pregnancies. Countries in SSA are currently facing the challenges of high birth rates that may be attributable to inadequate access and use of contraceptive methods. In response to this, the governments of these countries are focusing on the need for mass communication campaigns to encourage the use of contraceptives. The empirical evidence documented in some studies although limited in scope, geographical boundaries, and in some cases statistical analysis rigor has shown that exposure to family planning (FP) information remains critical if we intend to increase contraceptive use among women of reproductive age^13–15^. In addition, the investment made in FP education over the years has been enormous. For instance, the direct and indirect annual cost (program support, information and education on family planning, construction and maintenance of facilities, and supply chain management) of providing modern contraceptive services to 671 million users in developing regions was estimated to be US$6.3 billion^14^. Despite this huge investment in FP campaign messages, there is a paucity of evidence regarding the effectiveness of FP messages on contraceptive use in sub-Saharan Africa. In Africa, some studies have estimated the pooled prevalence of contraceptive use and determined associated factors among reproductive women using nationally stratified surveys^16,17^, however, none of them have assess the effectiveness of FP messages on contraceptive use. Using the most recent demographic and health survey data, we determine the prevalence trends and quantify the impact of exposure to FPM on CU among women of reproductive age in SSA.

## Methods

This study followed the standard guidelines for reporting observational studies using the Strengthening the Reporting of Observational Studies in Epidemiology (STROBE).

### Data source

This study utilized secondary data from the most recent and available Demographic and Health Survey (DHS) conducted in 26 SSA countries between 2013 and 2019 (Supplementary Table 1). The DHS is a nationally representative household survey with similar data collection instruments and study designs conducted in LMICs with the primary goal of generating estimates for indicators that are comparable across the sub-region. The DHS provides data for a wide range of monitoring and impact evaluation indicators in the areas of population, health, and nutrition. Specifically, the DHS collects data on family planning (knowledge and use of contraceptives), maternal health (antenatal, delivery, and postnatal care), household wealth, parity, education, place of residence, and demographics, amongst other variables with sample sizes (usually between 5000 and 30,000 households) and typically are conducted in every 5 years, to allow comparisons over time. The survey employs a multi-stage stratified cluster sampling design where the index country is stratified into distinct geographical regions or provinces during the first phase of the design. The first phase of sampling involves the random sampling of clusters or enumeration areas (EA) using probability proportional to the size of the EA and the subsequent sampling of a fixed number of households within each of the sampled enumeration areas using a systematic random sampling approach. A complete household listing was carried out to update the sampling frame before the random sampling of households. Trained field data collectors were assigned to these sampled enumeration areas for the household survey. Details on the study design and procedures for data collection have been published elsewhere^18^.

The DHS data is publicly available upon reasonable written request at the DHS website (https://dhsprogram.com/data/available-datasets.cfm).

All standard DHS surveys have been reviewed and approved by ICF Institutional Review Board (IRB). Additionally, country-specific DHS survey protocols are reviewed by the ICF IRB and typically by an IRB in the host country (https://dhsprogram.com/Methodology/Protecting-the-Privacy-of-DHS-Survey-Respondents.cfm). This study did not require country-specific ethical approval since we only analyze secondary data from the DHS program that has obtained ethical approval for all countries for the different survey years and all study participants have been de-identified.

DHS is one of the few nationally representative household surveys with very high response rate (> 95%). Because of this high response rate, we assumed that missing data will be missing completely at random. This implies that there would be no systematic differences in the observed characteristics between participants with missing data and those with complete data.

### Outcome variable

The primary outcome measure in this study was contraceptive use. Contraceptive use as defined by DHS was among women of reproductive age who currently use any standard method of contraceptive (traditional or modern). Contraceptive use was classified as a binary variable that takes the value of 1 if the woman is currently using a traditional or modern contraception method and a value of 0 if otherwise. The modern methods include women who use female sterilization (tubal ligation, laparotomy, voluntary surgical contraception), male sterilization (vasectomy, voluntary surgical contraception), the contraceptive pill (oral contraceptives), intrauterine contraceptive device (IUD), injectables (Depo-Provera), implant (Norplant), female condom, the male condom (prophylactic, rubber), diaphragm, contraceptive foam and contraceptive jelly, lactational amenorrhea method (LAM), standard days method (SDM) and country-specific modern methods. Respondents mentioned other modern contraceptive methods (including cervical cap, contraceptive sponge, and others), but do not include abortions and menstrual regulation^19^.

### Primary exposure

Exposure to FPM was defined as individual women of reproductive age who heard or saw FPM on the radio, on television, in a newspaper or magazine, or on a mobile phone in the past few months^19^.

### Confounders

Variables considered as possible confounders were selected based on an extensive literature review of factors that could potentially influence access to FPM and contraceptive use among women of reproductive age*.* The following variables were accounted for in all the multivariable models: the age of the household head (categorized as ≤ 29, 30–39, 40–49, 50–59, and 60+), sex of the household head (male or female), household wealth Index (poorest, poorer, middle, richer, richest), place of residence (rural or urban), religion (Islam, Christian or Others), respondent age (15–19, 20–29, 30–39, 40–49), marital status (widowed, never married, married or divorced), educational level (no formal education, primary, secondary, higher), currently working (no, yes), children ever born (no child, 1 child, 2 children, 3 + children)^20,21^. These variables have been found to either increase contraceptive use, exposure to family planning messages or both.

### Statistical analysis

#### Assessing trend and factors associated with contraceptive use

We explored the trend of FPM and CU between 2013 and 2019 using tools from time series line graphs and estimated the weighted prevalence of FPM and CU over the period by adjusting for sampling weight for all point and interval estimates including regression models. Factors contributing to CU and FPM were assessed using the Poisson regression model with a cluster-robust standard error that generates prevalence ratios and their respective confidence intervals. Sensitivity analysis of the point estimates and corresponding confidence interval (CI) was conducted using the multivariable binary logistic regression model that reports odds ratio and CI. The Poisson model was preferred to the logistic regression model as the odds ratio may overestimate the prevalence ratio, the measure of choice in cross-sectional studies^22^.

#### Assessing impact of family planning messages on contraceptive use

Augmented inverse-probability weighting (AIPW) was used to estimate the average treatment effect of FPM from cross-sectional data. The AIPW estimator is classified among the estimators with the doubly-robust property as it combines aspects of regression adjustment and inverse-probability-weighted methods to reduce bias associated with the impact estimate. The model accounted for sampling weight and used cluster-robust standard errors to address the methodological challenges (stratification, clustering, weighting) associated with complex survey design. Since different impact estimation procedures may lead to slightly different impact estimates especially when the data originates from crossectional studies instead of the more rigorous experimental design, sensitivity analysis of the impact estimate was conducted using endogenous treatment effect models, inverse probability weighting, propensity scores, and nearest-neighbor matching techniques. Estimating the impact of an intervention, program or policy becomes difficult due to endogeneity. For instance, genetic predisposition, personal values, conservative lifestyle, religious beliefs, and other unmeasured confounders may simultaneously affect exposure to family planning messages and utilization of contraception^13^. The standard regression models (e.g., Poisson, Negative Binomial, binary logistic, probit, and ordinary least square assume that these unmeasured covariates do not correlate with both the outcome measure (contraceptive use) and exposure to FPM. This assumption is largely violated in the context of observational data where both the outcome and exposure are usually measured at the same time and may correlate with unobserved confounders. We anticipated these problems, and as part of the sensitivity analyses that were conducted, we used endogenous treatment regression models to address endogeneity. Having radio or television was used as the instrumental variable since it met the exclusion restriction criteria recommended for instrumental variable regression analysis (that is, having a radio or television sets influence the ability to listen to FPM directly, it does not influence the use of contraceptives directly, but only through the family planning message and we assume that it is not influenced by other factors).

All statistical analyses were conducted using Stata version 17 (StataCorp, College Station, Texas, USA) and a p-value of less than 0.05 was considered statistically significant.

### Ethics approval and consent to participants

This is a secondary data analysis of publicly available data with de-identified participants' information.

## Results

### Characteristics of the study participants

The study involved 328,386 women of reproductive age (15–49 years) in SSA with an average of 30.5 years (standard deviation  8.9 years). Approximately 61% of the women lived in rural areas and 73% were married. About 30% of the women had no formal education. The sociodemographic characteristics of the women can be found in supplementary Table 2.

### Prevalence of contraceptive use and exposure to family planning messages

**Figure 1:**
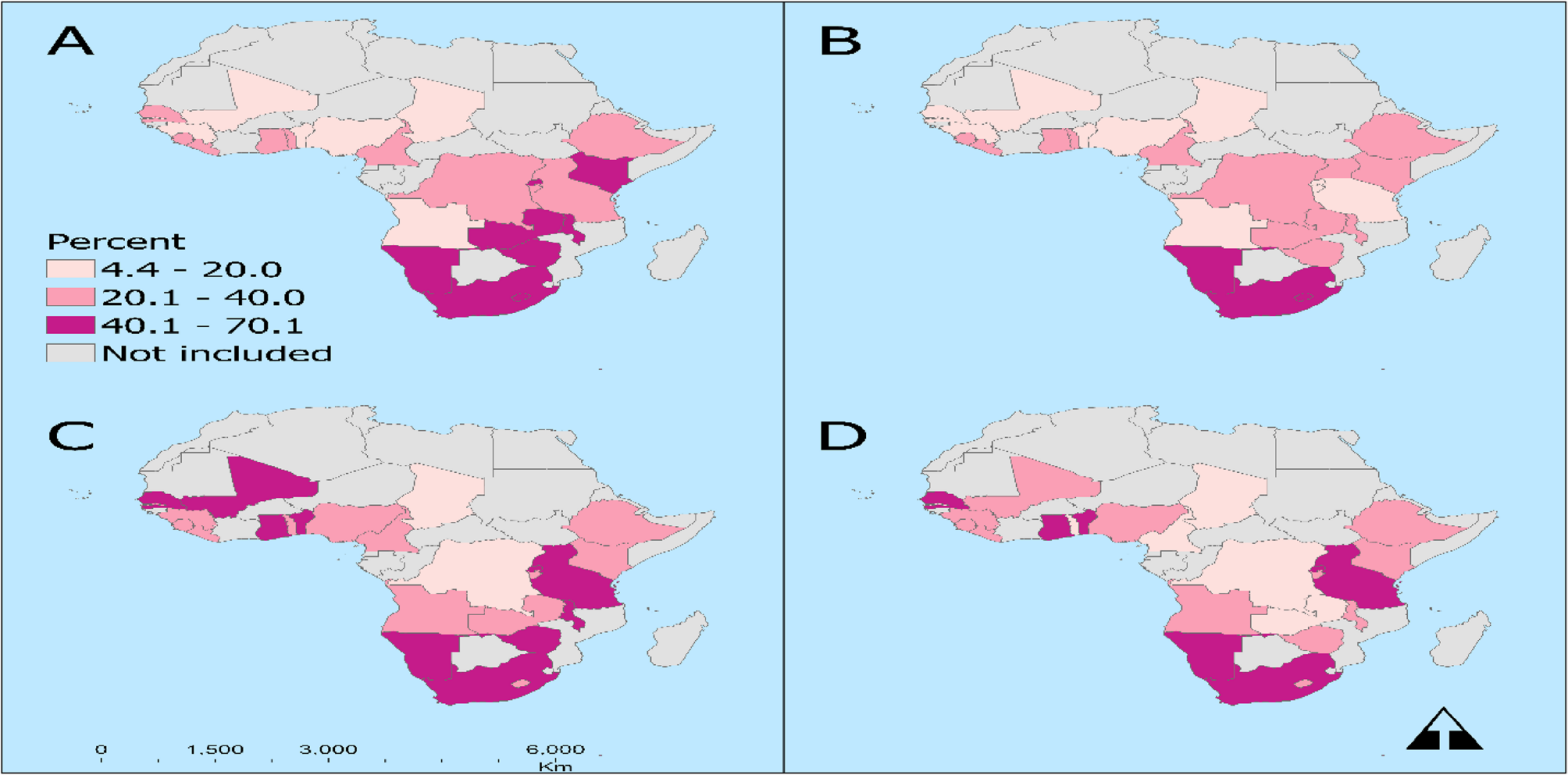
The geospatial distribution of contraceptive use and exposure to FP messages. Prevalence of contraceptive use by (**A**) general population, (**B**) adolescents and the exposure to family planning messages by (**C**) general population, and (**D**) adolescents among women of reproductive age in Sub-Saharan Africa, evidence from DHS surveys.

The overall prevalence of contraceptive use among women of reproductive age and adolescents in SSA between 2013 and 2019 was estimated as 31.1% (95%CI  30.6, 31.5) and 22.6% (95% CI 21.9–23.2) respectively (Table 1). The prevalence of contraceptive use was highest in Southern Africa [52.3% (95% CI 41.6–52.9)] and lowest in West Africa [0.4% (95% CI 19.9–20.9)]. By country, the Chad Republic recorded the lowest prevalence of contraceptive use [6.2% (95% CI 5.3–7.2)] with Zimbabwe recording the highest prevalence of contraceptive use [59.6% (95% CI 58.0–61.1)]. Approximately 39% (95% CI 38.8–39.4) and 32% (95% CI 31.36–32.94) of the women and adolescents were exposed to FPM in SSA between 2013 and 2019 respectively (Table 2). Eastern African countries were highly exposed to family planning messages [49.5%; 95% CI (48.3–50.6)] and the Central African countries were the least exposed to FPM [26.5%; 95% CI (25.4–27.7)]. By Country, Uganda recorded the highest exposure to FPM [70.1 (68.7–71.5)] and Chad recorded the least exposure to FPM [11.3 (9.9–12.9)]. The geospatial distribution of contraceptive use and exposure to FP messages can be found in Fig. 1.

**Table 1:** Trend of the prevalence of contraceptive use and exposure to family planning among women of reproductive age in sub-Saharan countries (2013–2019).

Sub-region	Country	Contraceptive use		Exposure to FP messages	
		General population	Adolescents	General population	Adolescents
		% [95% CI]	% [95% CI]	% [95% CI]	% [95% CI]
**Sub-Saharan Africa**	**Overall**	**31.1 [30.6–31.5]**	**22.56 [21.90–23.23]**	**38.90 [38.8–39.4]**	**32.14 [31.36–32.94]**
**Central Africa**	Angola	14.8 [13.0–16.8]	14.98 [12.36–18.05]	34.1 [31.0–37.4]	26.75 [23.44–30.34]
	Burundi	25.1 [23.9–26.3]	18.53 [15.21–22.38]	31.2 [29.9–32.6]	31.16 [26.97–35.68]
	Chad	6.2 [5.3–7.2]	4.46 [3.06–6.47]	11.3 [9.9–12.9]	11.74 [9.57–14.33]
	DR Congo	21.9 [19.9–24.1]	21.92 [18.65–25.58]	13.1 [11.0–15.5]	10.98 [8.78–13.64]
	Rwanda	52.5 [51.3–53.8]	18.35 [15.14–22.06]	54.5 [52.9–56.0]	47.68 [43.01–52.40]
	**Pooled**	**22.4 [21.5–23.3]**	**14.79 [13.35–16.35]**	**26.5 [25.4–27.7]**	**20.10 [18.54–21.75]**
**Eastern Africa**	Ethiopia	32.9 [30.7–35.3]	29.82 [25.06–35.05]	28.9 [26.0–31.9]	24.86 [20.02–30.43]
	Kenya	49.7 [48.6–50.8]	27.08 [24.50–29.81]	38.1 [37.3–38.9]	35.22 [32.29–38.26]
	Tanzania	37.0 [35.2–38.9]	19.82 [17.15–22.78]	68.7 [66.7–70.6]	65.09 [61.18–68.81]
	Uganda	35.4 [34.2–36.6]	21.71 [19.46–24.15]	70.1 [68.7–71.5]	66.22 [63.40–68.92]
	**Pooled**	**41.0 [40.1–41.8]**	**24.11 [22.66–25.63]**	**49.5 [48.3–50.6]**	**50.23 [48.25–52.21]**
**Southern Africa**	Lesotho	56.5 [54.6–58.3]	43.53 [39.33–47.83]	34.4 [32.3–36.6]	22.37 [18.89–26.28]
	Malawi	52.0 [50.8–53.2]	29.25 [27.11–31.49]	45.4 [43.7–47.1]	37.10 [34.58–39.70]
	Namibia	57.9 [56.3–59.5]	52.33 [47.85–56.77]	52.6 [50.1–55.0]	45.36 [40.41–50.41]
	South Africa	54.3 [52.6–56.0]	54.98 [50.13–59.74]	55.0 [52.5–57.5]	46.71 [41.47–52.02]
	Zambia	40.7 [39.4–42.0]	24.27 [21.58–27.17]	24.0 [22.1–26.0]	16.45 [14.14–19.04]
	Zimbabwe	59.6 [58.0–61.1]	37.02 [32.98–41.25]	44.5 [42.2–46.9]	31.82 [27.54–36.42]
	**Pooled**	**52.3 [41.6–52.9]**	**35.37 [33.91–36.86]**	**42.3 [41.3–43.2]**	**32.68 [31.16–34.24]**
**Western Africa**	Benin	16.4 [15.4–17.5]	13.79 [11.91–15.92]	47.4 [45.2–49.6]	41.41 [37.96–44.94]
	Cameroon	23.1 [21.2–25.1]	26.29 [22.87–30.04]	26.9 [24.6–29.3]	18.20 [15.62–21.10]
	Gambia	18.8 [17.5–20.2]	8.14 [5.41–12.08]	33.9 [31.5–36.3]	19.59 [15.63–24.26]
	Ghana	26.1 [24.5–27.8]	20.26 [16.30–24.89]	68.3 [65.7–70.7]	52.45 [47.15–57.70]
	Guinea	14.0 [12.3–15.8]	19.58 [16.17–23.51]	32.9 [30.0–35.9]	31.95 [36.37–36.37]
	Liberia	28.1 [26.0–30.3]	26.52 [23.30–30.01]	33.9 [31.1–36.9]	29.35 [25.03–34.07]
	Mali	17.9 [16.4–19.6]	13.99 [11.70–16.64]	40.7 [38.4–43.1]	39.18 [5.43–43.07]
	Nigeria	17.0 [16.0–18.1]	8.29 [7.01–9.78]	36.5 [35.0–38.1]	22.19 [20.10–24.43]
	Senegal	26.1 [24.1–28.1]	10.38 [7.83–13.66]	60.3 [57.7–62.9]	43.41 [36.75–50.33]
	Sierra Leone	26.9 [25.7–28.2]	37.13 [34.15–40.21]	31.8 [29.2–34.5]	29.07 [25.97–32.38]
	Togo	21.9 [20.4–23.5]	24.98 [20.98–29.47]	22.1 [20.2–24.0]	19.26 [16.13–22.83]
	**Pooled**	**20.4 [19.9–20.9]**	**19.35 [18.36–20.37]**	**38.2 [37.4–39.1]**	**29.73 [28.57–30.91]**
**Year**	2013	30.6 [29.1–32.0]	29.5 [27.0–32.1]	24.9 [23.3–26.6]	20.5 [18.4–22.9]
	2014	34.9 [33.8–36.1]	20.3 [18.7–22.0]	34.8 [33.6–35.9]	27.6 [25.8–29.5]
	2015	41.1 [40.0–42.2]	23.8 [22.3–25.3]	47.6 [46.3–48.8]	39.5 [37.6–41.3]
	2016	35.1 [34.2–36.0]	28.1 [26.2–30.1]	47.3 [45.9–48.7]	49.5 [47.0–51.9]
	2017	16.4 [15.4–17.5]	13.8 [11.9–15.9]	47.4 [45.2–49.6]	41.5 [37.9–44.9 [
	2018	21.3 [20.6–22.0]	16.7 [15.5–18.0]	33.2 [32.3–34.2]	24.2 [22.9–25.7]
	2019	31.4 [30.4–32.3]	26.5 [24.7–28.3]	41.5 [40.2–42.8]	31.7 [29.6–33.8]

Values in bold highlight the estimates at the regional block level.

**Table 2:** Impact of exposure to family planning messages on contraceptive use among women in their reproductive year in Sub-Saharan Countries, evidence from DHS study.

Sub-region	Country	AIPW	ETE	IPW	NNMatch	PSMatch
		aβ [95%CI]	aβ [95%CI]	aβ [95%CI]	aβ [95%CI]	aβ [95%CI]
**Sub-Saharan Africa**	**Overall impact**	0.071 [0.067–0.0744]***	0.053 [0.038–0.068]***	0.063 [0.057–0.068]***	0.057 [0.053–0.061]***	0.058 [0.054–0.062]***
**Central Africa**	Angola	0.044 [0.029–0.058]***	0.080 [0.046–0.114]***	0.063 [0.036–0.091]***	0.051 [0.035–0.068]***	0.040 [0.025–0.055]***
	Burundi	0.031 [0.014–0.048]***	0.145 [0.049–0.241]**	0.040 [0.018–0.061]***	0.013 [0.013–0.050]***	0.028 [0.009–0.047]**
	Chad	0.085 [0.062–0.109]***	0.126 [0.091–0.162]***	0.123 [0.076–0.168]***	0.088 [0.056–0.012]***	0.099 [0.067–0.131]***
	DR Congo	0.028 [0.003–0.0530]*	0.051 [−0.005 to 0.107]	0.031 [−0.001 to 0.071]	0.035 [0.008–0.063]*	0.016 [−0.010 to 0.041]
	Rwanda	0.007 [−0.011 to 0.025]	0.087 [−0.016 to 0.192]	0.006 [−0.013 to 0.026]	0.010 [−0.010 to 0.029]	0.009 [−0.011 to 0.030]
	**Pooled**	0.067 [0.057–0.077]***	0.046 [0.029–0.064]***	0.074 [0.057–0.090]***	0.046 [0.035–0.057]***	0.046 [0.034–0.058]***
**East Africa**	Ethiopia	0.057 [0.033–0.081]***	−0.061 [−0.147 to 0.025]	0.016 [−0.023 to 0.056]	0.042 [0.014–0.071]**	0.047 [0.016–0.078]**
	Kenya	0.059 [0.034–0.084]***	0.135 [−0.007 to 0.0277]	−0.0001 [−0.041 to 0.04]	0.066 [0.037–0.095]***	0.037 [0.007–0.068]*
	Tanzania	0.053 [0.034–0.072]***	0.172 [0.019–0.326]*	0.032 [0.010–0.054]**	0.040 [0.018–0.062]***	0.050 [0.027–0.072]***
	Uganda	0.017 [0.00–0.033]*	−0.020 [−0.168 to 0.127]	0.014 [−0.008 to 0.038]	0.013 [−0.006 to 0.031]	0.010 [−0.010 to 0.029]
	**Pooled**	0.042 [0.032–0.051]***	**0.028 [−0.026 to 0.083]**	**0.013 [−0.002 to 0.028]**	0.040 [0.029–0.052]***	0.030 [0.018–0.043]***
**Southern Africa**	Lesotho	0.047 [0.017–0.079]**	0.008 [−0.285 to 0.301]	0.046 [0.010–0.080]**	0.047 [0.013–0.081]**	0.042 [0.008–0.077]*
	Malawi	0.019 [0.005–0.033]**	0.008 [−0.069 to 0.086]	0.012 [−0.003 to 0.029]	0.018 [0.003–0.033]*	0.017 [0.002–0.032]*
	Namibia	0.018 [−0.005 to 0.041]	0.046 [−0.179 to 0.270]	0.018 [−0.009 to 0.046]	0.017 [−0.009 to 0.043]	0.015 [0.012–0.042]
	South Africa	0.030 [0.006–0.039]*	0.058 [−0.446 to 0.562]	0.022 [−0.011 to 0.055]	0.037 [0.011–0.063]**	0.028 [0.001–0.055]*
	Zambia	0.017 [−0.006 to 0.039]	0.0001 [−0.15 to 0.15]	0.028 [0.001–0.054]*	0.015 [−0.011 to 0.041]	0.023 [−0.003 to 0.049]
	Zimbabwe	0.026 [0.005–0.047]*	0.177 [0.065–0.290]**	0.023 [−0.003 to 0.049]	0.012 [−0.011 to 0.035]	0.014 [−0.011 to 0.039]
	**Pooled**	0.022 [0.013–0.030]***	0.062 [0.001–0.124]*	0.019 [0.008–0.029]***	0.020 [0.011–0.029]***	0.022 [0.012–0.032]***
**West Africa**	Benin	0.044 [0.032–0.057]***	0.032 [-0.087–0.152]	0.040 [0.025–0.055]***	0.042 [0.028–0.056]***	0.044 [0.030–0.058]***
	Cameroon	0.067 [0.044–0.090]***	0.056 [0.022–0.089]***	0.073 [0.047–0.101]***	0.083 [0.056–0.0111]***	0.060 [0.032–0.088]***
	Gambia	0.021 [0.004–0.037]*	0.601 [0.570–0.631]***	0.009 [−0.014 to 0.032]	0.026 [0.006–0.045]**	0.024 [0.004–0.043]*
	Ghana	0.033 [0.012–0.053]**	0.134 [0.031–0.236]**	0.033 [0.005–0.061]*	0.035 [0.011–0.059]**	0.022 [−0.003 to 0.047]
	Guinea	0.051 [0.036–0.065]***	0.080 [0.005–0.155]*	0.050 [0.026–0.073]***	0.052 [0.036–0.068]***	0.047 [0.031–0.063]***
	Liberia	0.010 [−0.012 to 0.032]	−0.025 [−0.159 to 0.110]	0.010 [−0.012 to 0.032]	0.005 [−0.019 to 0.029]	0.013 [−0.012 to 0.037]
	Mali	0.024 [0.009–0.039]**	0.451 [0.345–0.556]***	0.025 [0.004–0.046]*	0.023 [0.006–0.041]**	0.021 [0.004–0.038]*
	Nigeria	0.017 [0.009–0.025]***	0.034 [0.013–0.055]***	0.016 [0.004–0.027]**	0.018 [0.009–0.026]***	0.017 [0.008–0.026]***
	Senegal	0.066 [0.045–0.087]***	0.253 [0.152–0.353]***	0.053 [0.021–0.085]***	0.06 [0.040–0.087]***	0.064 [0.039–0.089]***
	Sierra Leone	0.052 [0.034–0.070]***	0.026 [−0.013 to 0.066]	0.052 [0.030–0.075]***	0.052 [0.033–0.072]***	0.041 [0.021–0.062]***
	Togo	0.053 [0.031–0.075]***	0.029 [−0.03 to 0.084]	0.054 [0.027–0.080]***	0.065 [0.040–0.091]***	0.045 [0.021–0.069]***
	**Pooled**	0.042 [0.038–0.047]***	0.050 [0.032–0.068]***	0.042 [0.035–0.048]***	0.041 [0.036–0.046]***	0.040 [0.035–0.045]***

Analysis adjusted for; the age of household head, sex of household head, wealth index, place of residence, religion, respondent age, marital status, educational level, currently working, and the number of children ever born.

*DHS*  Demographic Health Survey, *AIPW* augmented inverse probability weighting, *ETE* endogenous treatment effects, *IPW* inverse probability weighting, *NNMatch* nearest neighborhood matching (1:1), *PSMatch* propensity score matching (1:1), *aβ* adjusted coefficient estimate, *CI* confidence interval.

P-value notation: *p-value < 0.05, **p-value < 0.01, ***p-value < 0.001.

Values in bold highlight the estimates at the regional block level.

### Trend analysis of contraceptive use and exposure to family planning messages

**Figure 2:**
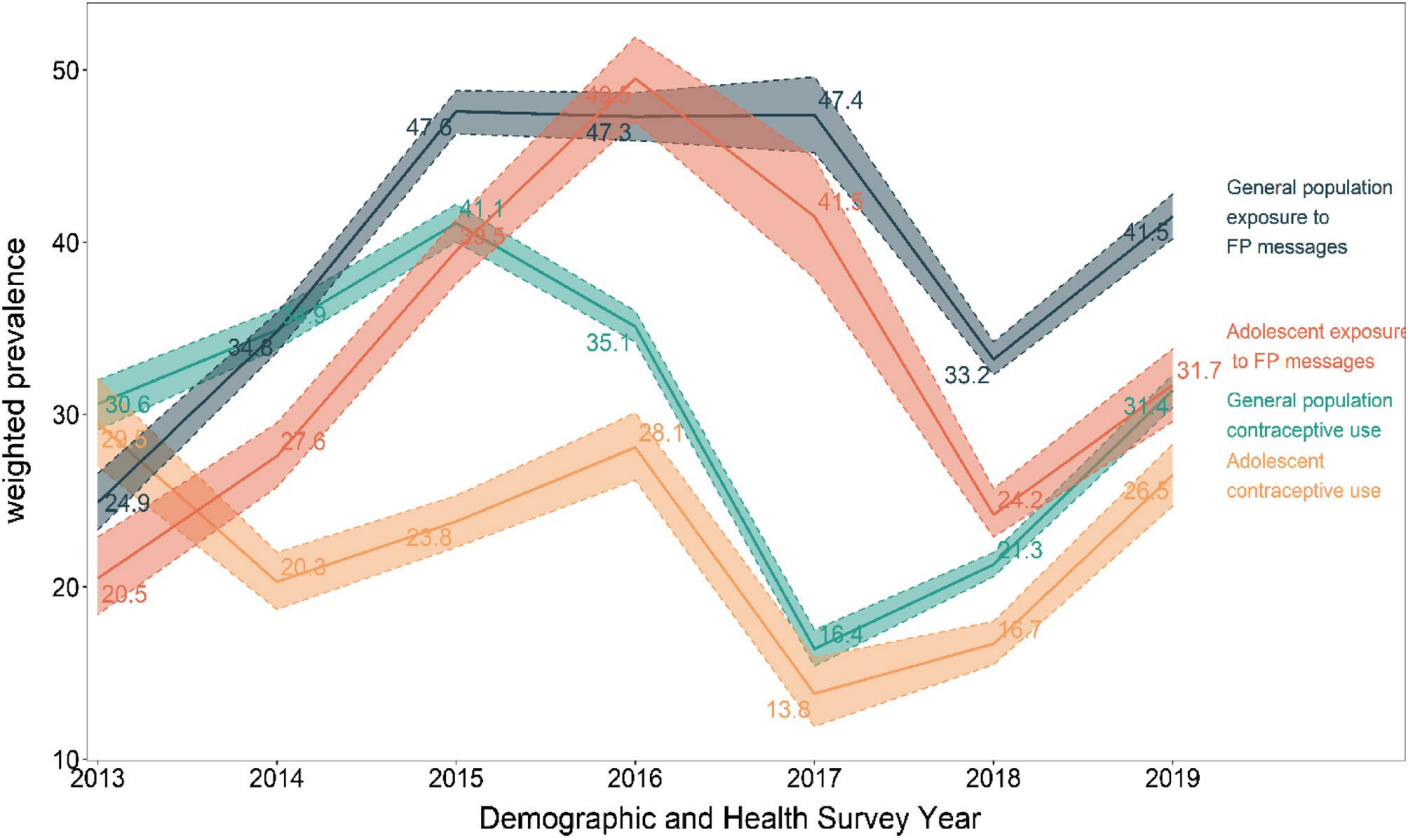
Trend of contraceptive use and exposure to family planning among women in their reproductive year in sub-Saharan countries, evidence from DHS study. *FP* family planning.

The trend analysis showed that the prevalence of contraceptive use among women of reproductive age and adolescence fluctuated between 2013 and 2019 but increased marginally between 2015 and 2017. Contraceptive use among women of reproductive age increased between 2013 and 2015 and declined between 2015 to 2017. Among adolescents, CU increased between 2013 and 2015 and remained fairly constant between 2015 and 2017 but declined between 2017 and 2018 before increasing marginally in 2019 (Fig. 2). There was a positive correlation between exposure to FPM and CU as a higher prevalence of CU was associated with higher exposure to FPM and vice versa (Fig. 2).

### Factors associated with access to family planning message

The following factors were found to be associated with access to family planning messages: age of the respondent, female household heads, higher socio-economic status measured via household wealth, living in urban areas, religion, marital status, higher education level, parity and women who were currently working at the time of the survey were found to be associated with a higher prevalence of access to FPM (Supplementary Table 3).

### Factors associated with contraceptive use

The results from the multivariable Poisson regression model showed that the age of the household head, sex of household head, higher socio-economic status measured via household wealth, living in urban areas, religion, marital status, higher education level, parity, and exposure to FPM were found to be associated with contraceptive use (Supplementary Table 4).

### Impact of exposure to family planning information messages on contraceptive use among women of reproductive age: evidence from DHS study

Table 2 shows the results from the augmented inverse probability weighting with regression adjustment and sensitivity analysis of the impact estimate among women of reproductive age. Exposure to FPM increased contraceptive use by 7.1 percentage points (pp) (95% CI  6.7, 7.4) among women of reproductive age in SSA. The impact of FPM on contraceptive use was highest in Central Africa [6.7 pp; 95% CI: (5.7–7.7) and lowest in Southern Africa (2.2 pp; 95% CI: (1.3–3.0)]. Cameroon recorded the highest impact of FPM on contraceptive use (6.7 pp; 95% CI: (4.4–9.0)] but exposure to FPM did not have a statistically significant effect on contraceptive use in Rwanda, Namibia, and Liberia.

### Impact of exposure to family planning information messages on contraceptive use among adolescents: evidence from DHS study

Table 3 shows the results from the augmented inverse probability weighting with regression adjustment and sensitivity analysis of the impact estimate of FPM among adolescents. Exposure to FPM increased CU by 6.0 percentage points (pp) (95% CI  5.0, 8.0) among adolescents in SSA. The impact of FPM on CU was highest in Southern Africa [7.0 pp; 95% CI: (4.0–9.0)] and lowest in Eastern Africa [2.0 pp; 95% CI: (−0.00, −3.0)]. Cameroon recorded the highest impact of FPM on contraceptive use among adolescents [impact estimate  17.0 pp; 95% CI: (9.0–25.0)].

**Table 3:** Impact of exposure to family planning information messages on contraceptive use among adolescents aged 15–19 years in Sub-Saharan Countries, evidence from DHS study.

Sub-region	Country	AIPW	ETE	IPW	PSM
		aβ [95%CI]	aβ [95%CI]	aβ [95%CI]	aβ [95%CI]
**Sub-Saharan Africa**	**Overall impact**	0.06 [0.05–0.08]***	0.03 [0.02–0.05]***	0.05 [0.03–0.06]***	0.06 [0.05–0.07]***
**Central Africa**	Angola	0.04 [0.01–0.08]*	0.08 [−0.05 to 0.12]***	0.05 [0.00–0.10]*	0.04 [0.01–0.08]**
	Burundi	0.08 [0.01–0.16]*	0.77 [0.72–0.81]***	0.09 [0.02–0.16]**	0.09 [0.01–0.16]*
	Chad	0.09 [0.02–0.16]**	0.13 [0.10–0.16]***	0.09 [0.02–0.15]**	0.12 [0.03–0.22]**
	DR Congo	0.05 [−0.03 to 0.13]	0.08 [0.03–0.13]***	−0.00 [−0.09 to 0.08]	0.08 [−0.01 to 0.17]
	Rwanda	0.00 [−0.06–0.07]	−0.10 [−0.21 to 0.01]	−0.01 [−0.07 to 0.05]	−0.03 [−0.10 to 0.04]
	**Pooled**	0.08 [0.05–0.11]***	0.12 [0.10–0.14]***	0.05 [0.02–0.09]***	0.09 [0.06–0.13]***
**Eastern Africa**	Ethiopia	0.08 [0.01–0.15]*	−0.01 [−0.09 to 0.07]	0.01 [−0.10–0.12]	0.07 [−0.01 to 0.15]
	Kenya	0.05 [0.01–0.09]**	−0.02 [−0.05 to 0.02]	0.04 [−0.01–0.09]	0.04 [0.00–0.08]*
	Tanzania	0.02 [−0.02 to 0.07]	−0.55 [−0.78 to 0.32]***	0.01 [−0.05 to 0.07]	0.03 [−0.02 to 0.08]
	Uganda	0.01 [−0.02 to 0.05]	0.18 [0.10 to 0.26]***	−0.01 [−0.05 to 0.04]	0.01 [−0.03 to 0.05]
	**Pooled**	0.02 [−0.00 to 0.04]	−0.05 [−0.07 to 0.03]***	−0.01 [−0.03 to 0.02]	0.02 [0.00–0.04]*
**Southern Africa**	Lesotho	0.09 [−0.02 to 0.19]	0.01 [−1.06 to 1.07]	0.03 [−0.08 to 0.14]	0.09 [−0.03 to 0.21]
	Malawi	0.02 [−0.02 to 0.06]	−0.03 [−0.29 to 0.22]	0.00 [−0.04 to 0.05]	0.01 [−0.02 to 0.05]
	Namibia	0.06 [−0.01 to 0.13]	−0.05 [−0.11 to 0.01]	0.08 [0.01–0.16]*	0.07 [−0.00 to 0.15]
	South Africa	0.01 [−0.08 to 0.09]	−0.07 [−0.20 to 0.06]	0.01 [−0.09 to 0.11]	−0.01 [−0.10 to 0.08]
	Zambia	0.05 [−0.01 to 0.12]	−0.09 [−0.18 to 0.00]*	0.09 [0.01–0.18]*	0.07 [0.00–0.14]*
	Zimbabwe	0.04 [−0.03 to 0.10]	−0.26 [−0.41 to 0.11]***	0.04 [−0.04 to 0.11]	0.05 [−0.03 to 0.13]
	**Pooled**	0.07 [0.04 to 0.09]***	−0.06 [−0.11 to 0.02]**	0.05 [0.02–0.08]***	0.07 [0.04–0.09]***
**Western Africa**	Benin	0.07 [0.04–0.11]***	0.39 [0.28–0.49]***	0.06 [0.02–0.10]**	0.07 [0.03–0.11]***
	Cameroon	0.17 [0.09–0.25]***	0.05 [0.02–0.08]***	0.08 [0.01–0.14]*	0.19 [0.12–0.25]***
	Gambia	0.02 [−0.03–0.08]	0.62 [0.59–0.65]***	0.02 [−0.05 to 0.09]	0.01 [−0.05 to 0.07]
	Ghana	0.01 [−0.04 to 0.07]	0.10 [0.02–0.19]**	−0.03 [−0.10 to 0.04]	0.00 [−0.06 to 0.07]
	Guinea	0.11 [0.06–0.17]***	0.48 [0.41–0.55]***	0.11 [0.05–0.18]***	0.11 [0.06–0.16]***
	Liberia	0.03 [−0.04 to 0.09]	−0.04 [−0.11 to 0.02]	0.01 [−0.07 to 0.09]	0.02 [−0.04 to 0.08]
	Mali	0.05 [0.00–0.09]*	0.02 [-0.01 to 0.05]	0.02 [−0.02 to 0.07]	0.06 [0.01–0.10]**
	Nigeria	0.03 [0.00–0.05]*	0.04 [0.03–0.06]***	0.02 [−0.01 to 0.05]	0.03 [0.00–0.05]*
	Senegal	0.09 [0.03–0.15]**	0.21 [0.14–0.28]***	0.07 [0.01–0.13]**	0.08 [0.02–0.14]**
	Sierra Leone	0.08 [0.01–0.14]*	0.01 [−0.02 to 0.05]	0.06 [−0.00 to 0.13]	0.07 [0.01–0.12]**
	Togo	0.14 [0.05–0.22]***	0.05 [0.00–0.09]*	0.13 [0.04–0.21]**	0.14 [0.05–0.23]***
	**Pooled**	0.06 [0.04–0.07]***	0.04 [0.03–0.05]***	0.04 [0.02–0.06]***	0.06 [0.04–0.07]***

Analysis adjusted for; the age of household head, sex of household head, wealth index, place of residence, religion, respondent age, marital status, educational level, currently working, and the number of children ever born.

*DHS* Demographic Health Survey, *AIPW* augmented inverse probability weighting, *ETE* endogenous treatment effects, *IPW* inverse probability weighting, *NNMatch* nearest neighborhood matching (1:1), *PSMatch* propensity score matching (1:1), *aβ* adjusted coefficient estimate, *CI* confidence interval.

P-value notation: *p-value < 0.05, **p-value < 0.01, ***p-value < 0.001.

Values in bold highlight the estimates at the regional block level.

## Discussion

This study assessed the prevalence, trends, and impact of exposure to FPM on contraceptive use among women of reproductive age in SSA and further conducted a sub-group analysis among the adolescent class of women using augmented inverse probability to treatment weighting with regression adjustment. Different sensitivity analyses were performed as a robustness check to confirm the results of augmented inverse probability to treatment weighting with regression adjustment. The empirical evidence presented in this manuscript allows us to draw four important conclusions. First, the prevalence of contraceptive use among women of reproductive age (general population aged from 15 to 49 years) and the adolescent sub-class largely varies among countries and geographic groupings in SSA and changes significantly over time. The high fluctuations in the prevalence of contraceptive use based on the trend analysis could be attributed to the variations in the level of intensity of family planning campaigns over the period, access and affordability of contraceptives in the sub-region.

The marginal increase in CU among the general population coupled with the declining CU among adolescents despite their increased exposure to FPM would indicate that regardless of exposure to messages, barriers to use persists.

Second, our final multivariable regression analyses showed that exposure to FPM does increase the likelihood of using contraceptive methods among women of reproductive age and adolescents sub-class in SSA although the effect size estimate varies by country and regional block. The regional and national diversity of SSA may play a key role in the diffusion of fertility regulating ideas and practices adopted by women^17^. Evidence of this is seen as contraceptive use among women of reproductive age and adolescents is higher in Southern Africa compared to Central, Eastern, and Western African countries. Eastern African countries are the most exposed to FPM this has been documented to be attributable to the government’s investments in improving access to SRH services through health insurance schemes, involvement of religious leaders in FP counseling and education, and introduction of health extension workers^23^. It is plausible that the structural and developmental changes such as that accompany urbanization such as the establishment of telecommunications networks and increased proliferation of cellular and smartphones could also be used to accelerate the spread of information on sexual and reproductive health. Policymakers and other stakeholders should intensify exposure to FPM using diverse media outlets such as television, radio, and print, and explore avenues for the appropriate use of electronic media.

Our third observation is that among the 26 SSA countries studied, there was a wide range of geographical differences in the prevalence of modern CU and exposure to FPM. Especially among the general population of women aged 15–49 years, the pattern of CU showed a decreasing array. Within the sub-regions, the lowest use of conceptive was among reproductive was observed among women residents in the WA region with approximately one-fifth prevalence rate. The need to address misinformation and fears of side effects as barriers to method use remains a critical area to be addressed in WA^24^. The prevalence of contraceptive use is a major public health concern in WA since the sub-region lagging in the use of contraceptives has been consistently so for more than two decades now^25^. Interestingly, the low utilization of contraceptives in WA is evident in the high total fertility rate compared with the general SSA region (5.1 versus 2.4)^26^.

Among the general population of women and adolescents, the high prevalence of CU and exposure to FPM in SSA occurred among women in Southern Africa and specifically Zimbabwe. Zimbabwean women have benefited from the strong post-independence encouragement of contraceptive use by their government^27^. For CU, approximately less than and a little more than one-twentieth of adolescents utilize contraceptives among adolescents and the general population respectively. The lowest prevalence of CU in Chad has also been confirmed by Ahinkorah et al., 2021^28^. Chad is at a disadvantage in both the use of contraceptives and exposure to FPM which needs urgent attention for improvement This calls for the adoption of new strategies to include adolescents in exposure to FPM programs since non-exposure to FPM directly translates into a high unmet need for FP among adolescents^29^. Communication is a vital mechanism connecting social factors and health outcomes.

Finally, we infer that exposure to FPM was found to be associated with CU among participants. Exposure to FPM was defined as hearing or seeing an FPM on the radio, television, in a newspaper or magazine, or on a mobile phone in the past few months. By using a counterfactual control group in this current study, the impact of exposure to FPM significantly increased the utilization of contraceptives in the SSA region. Findings envisage that the average conceptive use among women of reproductive age who are exposed to FPM significantly increased as compared with those who are not exposed. This finding corroborates the findings that exposure to FPM enhances the use of CU among reproductive-age women^30^.

Our study has provided empirical evidence to support the incessant calls for policymakers, external donor funding agencies, Civil Society and NGOs to prioritize and increase the resources for implementing family planning communication interventions in low-and middle-income countries. We proposed diverse country-specific policies, programs, and interventions that incorporate the different dynamics of socio-political, cultural, and other contextual factors that hinder access to family planning messages and the use of contraceptives in SSA.

Assessing the impact of health interventions poses a great challenge in situations where the data used for the analysis were from observational studies due to the problem of endogeneity (unobserved factors correlate with the treatment variable and the outcome measure of interest). Although a more rigorous statistical technique and sensitivity analysis of the impact estimates were conducted to generate an unbiased estimate of the program impact that addresses the problems of endogeneity, we believe that other unmeasured covariates (unobserved factors) such as health-related conditions, genetic predisposition, socio-cultural factors and area-specific inherent traditions in some part of SSA and many other factors may contribute to the observed change in the contraceptive use.

Notwithstanding these limitations, this impact evaluation study represents one of the few efforts to examine the effects of FPM on contraceptive use in SSA using data that originate from observation studies compared to the more preferred experimental study designs. It is the first study to assess the effect of family planning messages in SSA. In addition, the main outcomes were self-reported, which are subject to participants’ recall bias or socially desirable responses because the DHS asked the participant to recall over the past 30 days.

## Conclusion

Prevalence of CU and exposure to FPM varies significantly across countries in SSA and the exposure to family planning messages increased the use of contraceptive among women of reproductive age. Despite disparities observed, exposure to FPM has contributed to an increase in CU among women of reproductive age and the adolescent sub-class. We emphasized the need to implement policies that incorporate social-cultural and political support to encourage women to adopt contraceptive methods following exposure to messages. Funding for family planning education via print and electronic media should continue unabated.

## Supplementary Information

Supplementary Information.

## Data Availability

The datasets that were used in the study are publicly available on the DHS website (https://dhsprogram.com/data/available-datasets.cfm).

## Abbreviations

AIPW: Augmented inverse probability weighting
CU: Contraceptive use
DHS: Demographic and health survey
ETE: Endogenous treatment effect
FPM: Family planning messages
IPW: Inverse probability weighting
MM: Maternal mortality
PSM: Propensity score matching
SMI: Safe motherhood initiative
SSA: Sub-Saharan Africa
