# Supplementary material for: Exposure to family planning messages and contraceptive use among women of reproductive age in sub-Saharan Africa: a cross-sectional program impact evaluation study
Source: Sci Rep. 2022 Nov 7;12:18941. doi: 10.1038/s41598-022-22525-1 (PMC9640631; doi:10.1038/s41598-022-22525-1)
Supplement: Supplementary file 1 — Supplementary Information. [file 41598_2022_22525_MOESM1_ESM.docx]

**Exposure to family planning messages and contraceptive use among women of reproductive age in Sub-Saharan Africa: A cross-sectional program impact evaluation study**

**Supplementary Table 1: List of countries, survey year, and number of women that were used in the study**

| **Sub-region** | **Country** | **Survey year** | **Sample size** |
| --- | --- | --- | --- |
| **Sub-Saharan Africa** | **Overall** | **2013-2019** | **328386** |
| **Central Africa** | Angola | 2015 | 13072 |
|  | Burundi | 2016 | 12013 |
|  | Chad | 2014 | 15374 |
|  | DR Congo | 2013 | 16651 |
|  | Rwanda | 2019 | 10590 |
|  | **Pooled** |  | **67700** |
| **East Africa** | Ethiopia | 2016 | 11962 |
|  | Kenya | 2014 | 26461 |
|  | Tanzania | 2015 | 11285 |
|  | Uganda | 2016 | 15835 |
|  | **Pooled** |  | **65543** |
| **Southern Africa** | Lesotho | 2014 | 5689 |
|  | Malawi | 2015 | 21589 |
|  | Namibia | 2013 | 7992 |
|  | South Africa | 2016 | 7476 |
|  | Zambia | 2018 | 11882 |
|  | Zimbabwe | 2015 | 8130 |
|  | **Pooled** |  | **62758** |
| **West Africa** | Benin | 2017 | 13975 |
|  | Cameroon | 2018 | 11469 |
|  | Gambia | 2019 | 8824 |
|  | Ghana | 2014 | 8138 |
|  | Guinea | 2018 | 9162 |
|  | Liberia | 2019 | 7575 |
|  | Mali | 2018 | 9326 |
|  | Nigeria | 2018 | 35072 |
|  | Senegal | 2019 | 6495 |
|  | Sierra Leone | 2019 | 13965 |
|  | Togo | 2013 | 8384 |
|  | **Pooled** |  | **132385** |

**Supplementary Table 2: Sociodemographic characteristics of women in their reproductive year in Sub-Saharan Countries, evidence from DHS study**

| **Variable** | **Year** | | | | | | | **Total** | **Rao-Scot χ^2^** |
| --- | --- | --- | --- | --- | --- | --- | --- | --- | --- |
|  | **2013** | **2014** | **2015** | **2016** | **2017** | **2018** | **2019** |  |  |
|  | % | % | % | % | % | w% | % | % |  |
| **Age of household head** |  |  |  |  |  |  |  |  | 35.80*** |
| ≤29 | 16.6 | 18.1 | 22.1 | 19.8 | 14.8 | 12.3 | 11.1 | 16.4 |  |
| 30-39 | 28.4 | 31.6 | 30.6 | 31.0 | 29.3 | 27.9 | 25.1 | 29.2 |  |
| 40-49 | 27.8 | 26.6 | 25.4 | 26.1 | 27.1 | 27.8 | 26.5 | 26.8 |  |
| 50-59 | 14.5 | 13.7 | 12.7 | 12.3 | 14.8 | 16.8 | 17.5 | 14.6 |  |
| 60+ | 12.7 | 10.0 | 9.2 | 10.8 | 14.0 | 15.2 | 19.8 | 13.0 |  |
| **Sex of the household head** |  |  |  |  |  |  |  |  | 142.00*** |
| Male | 68.0 | 68.6 | 68.4 | 68.7 | 78.0 | 80.8 | 70.6 | 72.1 |  |
| Female | 32.0 | 31.4 | 31.6 | 31.3 | 22.0 | 19.2 | 29.4 | 27.9 |  |
| **Wealth Index** |  |  |  |  |  |  |  |  | 1.49 |
| Poorest | 17.9 | 16.7 | 18.7 | 19.4 | 17.8 | 18.4 | 19.1 | 18.3 |  |
| Poorer | 18.6 | 17.9 | 18.7 | 19.6 | 19.1 | 19.6 | 18.9 | 18.9 |  |
| Middle | 19.3 | 19.5 | 18.8 | 19.5 | 19.8 | 19.5 | 19.2 | 19.3 |  |
| Richer | 21.3 | 21.4 | 20.8 | 19.5 | 21.3 | 21.1 | 20.8 | 20.9 |  |
| Richest | 22.9 | 24.4 | 23.0 | 22.0 | 22.0 | 21.5 | 21.9 | 22.5 |  |
| **Place of residence** |  |  |  |  |  |  |  |  | 21.39*** |
| Urban | 43.6 | 37.7 | 36.4 | 27.4 | 41.8 | 42.0 | 46.0 | 39.0 |  |
| Rural | 56.4 | 62.3 | 63.6 | 72.6 | 58.2 | 58.0 | 54.0 | 61.0 |  |
| **Religion** |  |  |  |  |  |  |  |  | 471.48*** |
| Christian | 85.3 | 77.6 | 70.7 | 69.7 | 45.5 | 48.5 | 20.8 | 59.8 |  |
| Islam | 5.0 | 19.4 | 5.3 | 13.5 | 30.8 | 49.8 | 35.9 | 24.7 |  |
| Other+No religion | 9.5 | 2.7 | 6.6 | 1.1 | 23.6 | 1.7 | 0.3 | 3.4 |  |
| Missing | 0.2 | 0.3 | 21.4 | 15.8 | 0.0 |  | 43.1 | 12.0 |  |
| **Respondent age** |  |  |  |  |  |  |  |  | 21.43*** |
| 15-19 | 11.5 | 9.7 | 13.1 | 8.3 | 11.5 | 10.8 | 9.7 | 10.6 |  |
| 20-29 | 40.0 | 40.1 | 39.7 | 39.2 | 40.8 | 38.1 | 36.2 | 38.9 |  |
| 30-39 | 30.0 | 30.7 | 29.6 | 32.2 | 29.2 | 31.5 | 33.3 | 31.2 |  |
| 40-49 | 18.5 | 19.5 | 17.7 | 20.2 | 18.5 | 19.6 | 20.7 | 19.4 |  |
| **Marital status** |  |  |  |  |  |  |  |  | 85.64*** |
| Never married | 25.1 | 14.9 | 15.2 | 15.6 | 13.8 | 13.6 | 18.6 | 16.3 |  |
| Married | 65.5 | 72.8 | 70.8 | 72.0 | 80.0 | 78.8 | 73.2 | 73.4 |  |
| Divorced | 6.8 | 7.9 | 10.7 | 8.7 | 3.9 | 4.8 | 5.9 | 7.2 |  |
| Widowed | 2.6 | 4.4 | 3.3 | 3.7 | 2.3 | 2.8 | 2.4 | 3.2 |  |
| **Educational level** |  |  |  |  |  |  |  |  | 184.27*** |
| No education | 18.5 | 25.0 | 14.8 | 31.0 | 59.8 | 39.8 | 38.6 | 30.4 |  |
| Primary | 32.7 | 37.2 | 51.1 | 38.0 | 18.7 | 21.0 | 27.6 | 33.2 |  |
| Secondary | 43.7 | 29.8 | 30.3 | 24.8 | 19.4 | 31.3 | 28.8 | 30.3 |  |
| Higher | 5.1 | 7.9 | 3.9 | 6.3 | 2.1 | 7.9 | 5.0 | 6.1 |  |
| **Currently working** |  |  |  |  |  |  |  |  | 465.23*** |
| No | 32.1 | 30.7 | 33.6 | 37.1 | 19.7 | 34.9 | 33.3 | 31.1 |  |
| Yes | 67.6 | 43.7 | 66.4 | 62.9 | 80.3 | 65.1 | 66.7 | 62.5 |  |
| Missing | 0.3 | 25.7 |  |  |  |  |  |  |  |
| **Children ever born** |  |  |  |  |  |  |  |  | 28.49*** |
| No child | 17.8 | 14.0 | 13.4 | 12.2 | 15.6 | 14.6 | 14.5 | 14.3 |  |
| 1 Child | 17.9 | 17.1 | 17.8 | 17.0 | 14.8 | 15.4 | 18.3 | 17.0 |  |
| 2 Children | 15.6 | 16.8 | 16.8 | 16.1 | 14.0 | 14.5 | 16.4 | 15.9 |  |
| 3-4 Children | 22.9 | 24.8 | 26.4 | 24.0 | 25.5 | 24.3 | 25.5 | 24.8 |  |
| 5+ Children | 25.8 | 27.2 | 25.5 | 30.7 | 30.1 | 31.1 | 25.3 | 28.1 |  |

P-value Notation: ***p-value<0.001

**Supplementary Table 3: Factors associated with access to family planning information messages in Sub-Saharan Countries: evidence from DHS study**

| **Variable** | **General population** | | **Adolescents** | |
| --- | --- | --- | --- | --- |
|  | **PR[95%CI]** | **aPR[95%CI]** | **PR[95%CI]** | **aPR[95%CI]** |
| **Sex of household head** |  |  |  |  |
| Male | 1 | 1 | 1 | 1 |
| Female | 1.07[1.05-1.08]*** | 1.02[1.00-1.04]* | 1.07[1.02-1.11]** | 1.01[0.97-1.06] |
| **Wealth Index** |  |  |  |  |
| Poorest | 1 | 1 | 1 | 1 |
| Poorer | 1.34[1.29-1.38]*** | 1.26[1.21-1.30]*** | 1.28[1.19-1.38]*** | 1.21[1.11-1.31]*** |
| Middle | 1.68[1.63-1.74]*** | 1.48[1.42-1.54]*** | 1.58[1.46-1.71]*** | 1.40[1.28-1.52]*** |
| Richer | 2.12[2.05-2.19]*** | 1.71[1.65-1.78]*** | 1.96[1.82-2.12]*** | 1.63[1.49-1.78]*** |
| Richest | 2.66[2.57-2.76]*** | 1.88[1.80-1.96]*** | 2.44[2.26-2.62]*** | 1.83[1.67-2.01]*** |
| **Place of residence** |  |  |  |  |
| Rural | 1 | 1 | 1 | 1 |
| Urban | 1.59[1.55-1.62]*** | 1.11[1.08-1.14]*** | 1.49[1.42-1.56]*** | 1.09[1.02-1.15]** |
| **Religion** |  |  |  |  |
| Islam | 1 | 1 | 1 | 1 |
| Christian | 1.23[1.19-1.27]*** | 0.94[0.92-0.97]*** | 1.15[1.08-1.23]*** | 0.94[0.89-1.00] |
| Other+No religion | 0.97[0.91-1.03] | 0.96[0.91-1.01] | 1.05[0.91-1.21] | 1.03[0.91-1.18] |
| **Respondent age** |  |  |  |  |
| 40-49 | 1 | 1 | 1 ^ζ^ | 1 ^ζ^ |
| 15-19 | 0.84[0.82-0.86]*** | 0.76[0.75-0.79]*** | 1.18[1.13-1.23]*** ^η^ | 0.88[0.84-0.92]***^η^ |
| 20-29 | 1.04[1.02-1.05]*** | 0.92[0.90-0.93]*** |  |  |
| 30-39 | 1.05[1.03-1.06]*** | 0.98[0.97-0.99]*** |  |  |
| **Marital status** |  |  |  |  |
| Widowed | 1 | 1 | 1 | 1 |
| Never married | 1.31[1.27-1.36]*** | 1.15[1.11-1.20]*** | 5.69[1.81-17.9]** | 3.86[1.19-14.49]* |
| Married | 1.10[1.06-1.14]*** | 1.15[1.10-1.19]*** | 4.20[1.34-13.21]* | 3.54[1.09-11.44]* |
| Divorced | 1.11[1.07-1.16]*** | 1.05[1.01-1.09]* | 4.15[1.31-13.1]* | 3.10[0.95-10.09] |
| **Educational level** |  |  |  |  |
| No education | 1 | 1 | 1 | 1 |
| Primary | 1.53[1.49-1.57]*** | 1.49[1.45-1.53]*** | 1.47[1.37-1.59]*** | 1.41[1.30-1.55]*** |
| Secondary | 2.07[2.01-2.12]*** | 1.82[1.77-1.87]*** | 2.13[1.97-2.30]*** | 1.79[1.65-1.95]*** |
| Higher | 2.76[2.67-2.85]*** | 2.15[2.08-2.22]*** | 3.23[2.77-3.77]*** | 2.36[2.04-2.72]*** |
| **Currently working** |  |  |  |  |
| No | 1 | 1 | 1 | 1 |
| Yes | 1.23[1.21-1.26]*** | 1.22[1.20-1.24]*** | 1.05[1.00-1.09]* | 1.13[1.09-1.18]*** |
| **Children ever born** |  |  |  |  |
| 3+ Children | 1 | 1 | 1 | 1 |
| No child | 1.22[1.201.25]*** | 1.05[1.02-1.07]*** | 1.68[1.25-2.25]*** | 1.30[0.95-1.77] |
| 1 Child | 1.16[1.14-1.18]*** | 1.04[1.02-1.06]*** | 1.44[1.07-1.94]* | 1.30[0.96-1.78] |
| 2 Children | 1.18[1.16-1.20]*** | 1.05[1.03-1.06]*** | 1.33[0.98-1.81] | 1.26[0.91-1.73] |

Abbreviation: PR: Prevalence Ratio, aPR: adjusted Prevalence Ratio. NOTE: Superscript ζ denote reference category used for adolescents aged 15-17 years and η denote adolescents aged 18-19 years. P-value Notation: *p-value<0.05, **p-value<0.01, ***p-value<0.001.

**Supplementary Table 4: Association between exposure to family planning information messages and contraceptive use among women in their reproductive year in Sub-Saharan Countries, evidence from DHS study**

| **Variable** | **General population** | | **Adolescents** | |
| --- | --- | --- | --- | --- |
|  | **Poisson** | **Logistic** | **Poisson** | **Logistic** |
|  | aPR[95%CI] | aOR[95%CI] | aPR[95%CI] | aOR[95%CI] |
| **Exposure to FP messages** |  |  |  |  |
| No | 1 | 1 | 1 | 1 |
| Yes | 1.24[1.22-1.27]*** | 1.42[1.37-1.46]*** | 1.25[1.18-1.32]*** | 1.35[1.24-1.47]*** |
| **Sex of household head** |  |  |  |  |
| Male | 1 | 1 | 1 | 1 |
| Female | 0.93[0.92-0.95]*** | 0.90[0.87-0.93]*** | 1.01[0.95-1.07] | 1.01[0.93-1.10] |
| **Wealth Index** |  |  |  |  |
| Poorest | 1 | 1 | 1 | 1 |
| Poorer | 1.13[1.10-1.17]*** | 1.19[0.14-0.24]*** | 1.05[0.96-1.15] | 1.07[0.96-1.20] |
| Middle | 1.22[1.18-1.26]*** | 1.33[1.26-1.39]*** | 1.13[1.03-1.23]** | 1.17[1.03-1.32]** |
| Richer | 1.31[1.27-1.36]*** | 1.49[1.41-1.57]*** | 1.17[1.06-1.29]*** | 1.23[1.08-1.41]*** |
| Richest | 1.34[1.29-1.39]*** | 1.55[1.46-1.64]*** | 1.20[1.08-1.34]*** | 1.27[1.10-1.47]*** |
| **Place of residence** |  |  |  |  |
| Rural | 1 | 1 | 1 | 1 |
| Urban | 0.95[0.92-0.98]*** | 0.92[0.87-0.97]*** | 0.99[0.92-1.07] | 0.99[0.89-1.10] |
| **Religion** |  |  |  |  |
| Islam | 1 | 1 | 1 | 1 |
| Christian | 1.50[1.45-1.56]*** | 1.76[1.67-1.85]*** | 1.22[1.12-1.32]*** | 1.28[1.15-1.43] |
| Other+No religion | 1.28[1.19-1.36]*** | 1.38[1.26-1.51]*** | 1.16[0.98-1.37] | 1.20[0.96-1.49] |
| **Respondent age** |  |  |  |  |
| 40-49 | 1 | 1 | 1**^ζ^** | 1 |
| 15-19 | 1.17[1.12-1.22]*** | 1.32[1.15-1.32]*** | 0.86[0.81-0.91] **^***η^** | 0.81[0.75-0.87]*** |
| 20-29 | 1.29[1.26-1.33]*** | 1.50[1.43-1.57]*** |  |  |
| 30-39 | 1.28[1.25-1.31]*** | 1.42[1.42-1.53]*** |  |  |
| **Marital status** |  |  |  |  |
| Widowed | 1 | 1 | 1 | 1 |
| Never married | 2.25[2.11-2.41]*** | 3.14[2.88-3.44]*** | 1.36[0.69-2.68] | 1.55[0.66-3.63] |
| Married | 1.80[1.69-1.91]*** | 2.24[2.07-2.43]*** | 0.97[0.49-1.91] | 0.97[0.42-2.27] |
| Divorced | 1.53[1.43-1.63]*** | 1.73[1.59-1.90]*** | 1.08[0.54-2.15] | 1.12[0.47-2.67] |
| **Educational level** |  |  |  |  |
| No education | 1 | 1 | 1 | 1 |
| Primary | 1.75[1.70-1.81]*** | 2.10[2.02-2.18]*** | 1.99[1.75-2.60]*** | 2.17[1.87-2.51]*** |
| Secondary | 2.02[1.96-2.10]*** | 2.68[2.55-2.81]*** | 2.60[2.28-2.96]*** | 3.13[2.69-3.64]*** |
| Higher | 2.24[2.14-2.34]*** | 3.17[2.96-3.39]*** | 3.26[2.61-4.07]*** | 4.44[3.17-6.20]*** |
| **Currently working** |  |  |  |  |
| No | 1 | 1 | 1 | 1 |
| Yes | 0.99[0.96-1.00] | 0.98[0.95-1.01] | 1.03[0.97-1.09] | 1.04[0.96-1.12] |
| **Children ever born** |  |  |  |  |
| 3+ Children | 1 | 1 | 1 | 1 |
| No child | 0.48[0.46-0.50]*** | 0.31[0.29-0.34]*** | 0.87[0.61-1.21] | 0.80[0.53-1.22] |
| 1 Child | 0.71[0.69-0.73]*** | 0.57[0.54-0.60]*** | 1.32[0.93-1.85] | 1.44[0.95-2.18] |
| 2 Children | 0.89[0.87-0.91]*** | 0.84[0.82-0.89]*** | 1.21[0.85-1.71[ | 1.26[0.82-1.94] |

Abbreviation: PR: Prevalence Ratio, aPR: adjusted Prevalence Ratio, FP: Family Planning, aOR: adjusted odds ratio. NOTE: Superscript ζ denote reference category used for adolescents aged 15-17 years and η denote adolescents aged 18-19 years. P-value Notation: *p-value<0.05, **p-value<0.01, ***p-value<0.001
